# A Robust Noise Mitigation Method for the Mobile RFID Location in Built Environment

**DOI:** 10.3390/s19092143

**Published:** 2019-05-09

**Authors:** Changfeng Jing, Tiancheng Sun, Qiang Chen, Mingyi Du, Mingshu Wang, Shouqing Wang, Jian Wang

**Affiliations:** 1 School of Geomatics and Urban Spatial Informatics, Beijing University of Civil Engineering and Architecture, Beijing 100044, China; jingcf@bucea.edu.cn (C.J.); 2108521517012@stu.bucea.edu.cn (T.S.); dumingyi@bucea.edu.cn (M.D.); 2108521516015@stu.bucea.edu.cn (S.W.); 2108521517036@stu.bucea.edu.cn (J.W.); 2 Department of Geoinformation Processing, Faculty of Geo-Information Science and Earth Observation, University of Twente, P.O. Box 217, 7500 AE Enschede, The Netherlands; mingshu.wang@utwente.nl

**Keywords:** radio frequency identification (RFID), low-cost localization, noise mitigation, localization error

## Abstract

The exact location of objects, such as infrastructure, is crucial to the systematic understanding of the built environment. The emergence and development of the Internet of Things (IoT) have attracted growing attention to the low-cost location scheme, which can respond to a dramatic increasing amount of public infrastructure in smart cities. Various Radio Frequency IDentification (RFID)-based locating systems and noise mitigation methods have been developed. However, most of them are impractical for built environments in large areas due to their high cost, computational complexity, and low noise detection capability. In this paper, we proposed a novel noise mitigation solution integrating the low-cost localization scheme with one mobile RFID reader. We designed a filter algorithm to remove the influence of abnormal data. Inspired the sampling concept, a more carefully parameters calibration was carried out for noise data sampling to improve the accuracy and reduce the computational complexity. To achieve robust noise detection results, we employed the powerful noise detection capability of the random sample consensus (RANSAC) algorithm. Our experiments demonstrate the effectiveness and advantages of the proposed method for the localization and noise mitigation in a large area. The proposed scheme has potential applications for location-based services in smart cities.

## 1. Introduction

Location of infrastructure assets is crucial to a systematic understanding of the built environment, which is helpful for government administrators and urban planning authorities. Existing approaches for the exact location of infrastructure assets are image recognition [1], field survey [2,3], and light detection and ranging (LiDAR) information extraction [4], such as street view services [5], GPS-enabled video [6,7], geomagnetic [8], and other similar online technologies. Radio frequency identification (RFID)-based localization has advantages in sensing object without considering the massive occupation on the image and huge workload in fieldwork [9,10]. Therefore, it is more suitable for infrastructure assets localization in a complex urban surrounding. However, for applications such as in a large urban area or in the situation of an emergency, a low-cost and easy deployment localization scheme must be devised. Recently, a low-cost collaborative scheme with Global Navigation Satellite System (GNSS) and RFID was proposed [11].

Localization accuracy is an active research field [12,13]. The exact location of objects in the built environment is essential for monitoring, extracting, and analyzing spatial information. Hence, many methods were developed to improve the accuracy of RFID-based localizations. One type of approaches is to increase the number and density of the tags. Xiao et al. attached one more tag on the one object to discriminate the noise for improving accuracy [14]. However, these methods have significantly increased the cost. Besides the improvement of hardware, methods were developed from a software perspective, such as filtering algorithm [15], noise detection algorithm [16], clustering algorithm [17], and machine learning [18]. Many of these methods attempt to identify and correct the abnormal measurement or signal according to the localization principle. Therefore, we refer to them as logic-based approaches, while the machine learning methods predict error or measurements by engaging learning concept.

Random sample consensus (RANSAC) is a more robust noise mitigation method to yield very good results [13,19,20], which is widely used in building panel productions management [21], localization technologies [13,22], and other domains. Except for the RFID-based localization, RANSAC method was also used in GNSS-based and sound-based localization schema [23,24]. As for the application in RFID-based localization scenario, the most popular usage is to filter and refine the signal measurements for the higher accuracy. For example, the RANSAC method was used to estimate the parameters of RSSI-range localization models [22]. Researchers also used the RANSAC method to identify and filter outlier measurements [20]. Although RANSAC method is robust, many variants were developed aiming at increasing accuracy and efficiency. For example, some work aimed to optimize the process of model verification [25], the other works pursued to increase the efficiency of sampling process [26]. Furthermore, some summarized all variants to a universal framework [27], while others extended it to real-time scenario [28].

In general, most of existing noise mitigation methods suffer from the following drawbacks. First, the computation and preconditions of algorithms are complicated. Logic-based methods, such as particle filter method, suffer from high computational cost owing to repeatedly performance in sampling and filtering procedures [16]. Meanwhile, in machine learning methods, the training dataset is difficult to collect since the urban surrounding is a dynamic area with changed noise data. Second, the noise-detecting capacity of these methods cannot identify the most of noise data in complex urban scenario owing to the dynamic environment. For example, when automobiles or pedestrians obstruct the signal from the reader to tags, the noise may be changed sharply. Finally, these methods cannot directly be used in large area applications for the built environment since they are designed from local area localization with multiple tags or readers. However, in our low-cost localization scheme [11], only one moving RFID reader was deployed. Therefore, it is essential to develop a novel noise mitigation method for the low-cost localization scheme.

Therefore, in this paper, taking the aspects mentioned above into account, we proposed a new noise mitigation method based on RANSAC, supporting the low-cost localization scheme, lower computational consumption, powerful noise detection capability, and application in a large area. In our method, a proposed filtering algorithm as a preprocessing step is performed to detect and correct the anomaly of measurements first. Then, the carefully calibrated RANSAC model is used to achieve the location of the target tags.

The contributions of this paper are twofold. First, the RANSAC-based noise mitigation method was proposed for the infrastructure assets localization in the complex urban environment with a low-cost schema. Depending on the 50% powerful capability and sampling concept of the RANSAC model [29], our proposed method can achieve higher accuracy without adding extra devices or preparing prior training data. Second, a two-stage localization process integrating a delta filter algorithm and RANSAC-based noise mitigation method was developed to improve the localization accuracy in the built environment. Comparing the existing localization schema for the infrastructure assets, our scheme is low-cost and can achieve sufficient accuracy for the localization to the field of the built environment.

The rest of our paper is organized as follows. Section 2 covers related works. In Section 3, an overview of our previous low-cost localization scheme and the noise influence were introduced. Section 4 presents the novel noise mitigation method based on RANSAC. In Section 5, we design experiments to validate the effectiveness and accuracy of the proposed method. The result is posted in this section. Section 6 compares our method with existing similar methods and discusses the effectiveness. We conclude our work and provide paths for future work in Section 7.

## 2. Related Works

Location information is fundamental to build environment applications, such as infrastructure asset management [30] and infrastructure health monitoring [31,32]. The RFID-based method is a balanced scheme considering the cost, accuracy, and deployment.

### 2.1. RFID-Based Location

Through literature review, existing methods for RFID-based localization can be divided into three categories: Range-based, fingerprinting, and proximity [30,33]. The range-based method is widely used owing to its convenience and sufficient accuracy for most applications. However, the accuracy is heavily dependent on the device cost and the surrounding environment. Higher accuracy is achieved when devices that are more sophisticated and there are less signal interference sources in the surroundings. For the fingerprinting scheme, the prior knowledge and calibrated matching algorithms are necessary for higher accuracy. In some situation such as emergency applications, the prior collection of fingerprinting is more complicated and impractical [34]. On the other hand, the parameters of the fingerprinting matching algorithms have to be calibrated more carefully and change in different areas. For the proximity technology, it does not attempt to measure the distance from tag to RFID reader. Instead, it determines whether the tag is near RFID readers [35]. Therefore, the density of RFID readers determines the localization accuracy in this scheme. The shortcoming of the proximity method is low accuracy [15]. For the built environment applications such as urban infrastructure assets investigation, the low accuracy is not sufficient. A deployment with higher tag density means higher accuracy at the expense of a higher cost. To reduce the cost, reference tags at fixed point replace the RFID readers. However, in some special situation such as a museum, it is not feasible to deploy many reference tags.

In order to increase the suitability and practice in the large area, research on low-cost RFID-based scheme has been an active research field. Although the GNSS as a low-cost method can achieve enough accuracy for urban infrastructure assets, the shortcoming is the fragile signal for localization in density higher building areas. Integrating with other sensors can improve the accuracy [36] when the GNSS signal is weak, but it needs further investment for the expensive sensors. The ultra-wideband (UWB) is another widely used localization scheme for indoor and outdoor scenarios. However, it also has the cost disadvantage, and particularly, it needs more readers in a complex surrounding area [16,37]. Some research on reducing cost was published. The reference tags deployed at fix place is the most popular solution [38,39]. Other pieces of literature proposed tag matrix solutions [40] and virtual references replacing the physical reference tags [30]. However, more reference tags or careful calibration for virtual reference deployment are needed, which is impractical in a rapid developing built environment.

Although many localization scheme and low-cost solutions were developed, they cannot directly apply in the built environment for localization owing to the complex urban surrounding and accuracy requirement. For the RFID-based localization scheme, the accuracy of proximity technology is not enough for the built environment localization. The primary disadvantage of fingerprinting is the challenge of dynamic surrounding in an urban area, which requires the updating of prior fingerprinting. Therefore, a range-based scheme is more suitable for the built environment. Considering the cost of RFID-based scheme, the tags mounted on the infrastructure are necessary, which has little room for reducing cost. It is plausible to reduce the number of RFID readers. Indeed, a low-cost solution combining RFID and GNSS with only one mobile RFID reader was proposed [11]. However, there is more room to improve its accuracy.

### 2.2. RFID Noise Mitigation Methods

RFID-based localization was widely used in civil domains and built environment, such as production management for a panelized home prefabrication facility [21] and infrastructure assets operation [10]. The noise data is universally existing in the built environment. For noise mitigation, many approaches are developed. Chai et al. proposed the machine learning method based on support vector regression for the localization of facilities in severe noise environment [16]. The experiment in Liquefied Natural Gas (LNG) demonstrates the effectiveness and feasible for the robust detection and identification for the outliers. Using the RFID to collect the production data in production management, researchers developed the RANSAC model to clean the RFID raw data for the simulation model as input parameters [21].

In general, existing approaches for accuracy improvement can be classified into three categories: Filtering algorithms, noise detection approaches, and machine learning approaches. The first two classes identify and correct the abnormal measurement according to the localization principle and they belong to logic-based approaches. For the machine learning approach, it may predict the error or measurement with a learning concept.

The filtering algorithms are the most popular one which filters the abnormal signal or measurement [19]. Kalman filter and its variants, Bloom Filter, particle filter, and so on are among those famous filtering algorithms [15,41]. Abreu verified the capability of 15% accuracy improvement [42]. Xu et al. used a Gaussian filter to filter the abnormal Received Signal Strength (RSS) value [43]. Moreover, integrating several filter algorithms can enhance the localization accuracy. For example, Weighted Centroid Localization (WCL) and particle filter have been integrated to achieve high accuracy at a low computational cost [44].

Noise detection approaches are another popular solution for RFID-based localization. These methods include but are not limited to: Support Vector Regression (SVR) [16], clustering method [17,45], and Least Median of Squares (LMedS) [19]. Robust SVR combing robust learning algorithm with the SVR method was to enhance weight determination and overcome the computational complexity for tag [16]. Motamedi developed CMTL method based on cluster algorithm by clustering to group reference tag according to their spatial distribution for the movable tag localization [10]. As a conclusion, these methods attempt to identify the out-of-range measurement by regression models or other algorithms considering the spatial distribution, signal strength, or error threshold. However, under the development of localization technology, existing logic-based approaches have their limitations in modeling uncertainty and noise detection.

As a result, the intelligent learning methods were integrated to model the relationship or predict the uncertainty by machine learning methods [18]. The Hidden Markov Model is used to model the following sequential object sensor observations for fine-grained activity recognition [46]. Artificial Neural Network is engaged in establishing a classification model that can learn the relationship between the Received Signal Strength Indication (RSSI) and tag position accurately [47]. Extreme Learning Machine as a neural network is used to regress and classify for the RFID tag location [48]. However, there is an additional step required to prepare training data. The training dataset is more challenging to collect in urban environments, owing to the dynamic and complex environment.

In addition to the methods above, some novel methods were proposed to mitigate noise influence. For example, Liu et al. devised a method to estimate the spatial and temporal distribution of RFID tracking accuracy based on Geostatistical algorithm [49]. A confidence-based intersection method for trilateration based on probability concept was proposed to select the intersection point for higher location accuracy [13]. However, these methods are employed to reduce noise in a location scheme with multiple readers, which cannot directly apply in a low-cost solution with a mobile RFID reader. The possible reasons include the dynamic noise with a complex surrounding, the computational complexity in parameter estimation [19], or overfitting in regression [16]. Therefore, devising a method for complex surrounding support lower computational complexity is a pressing challenge.

## 3. Mobile Localization and Problem Formulation

### 3.1. Mobile Localization Method

**Figure 1:**
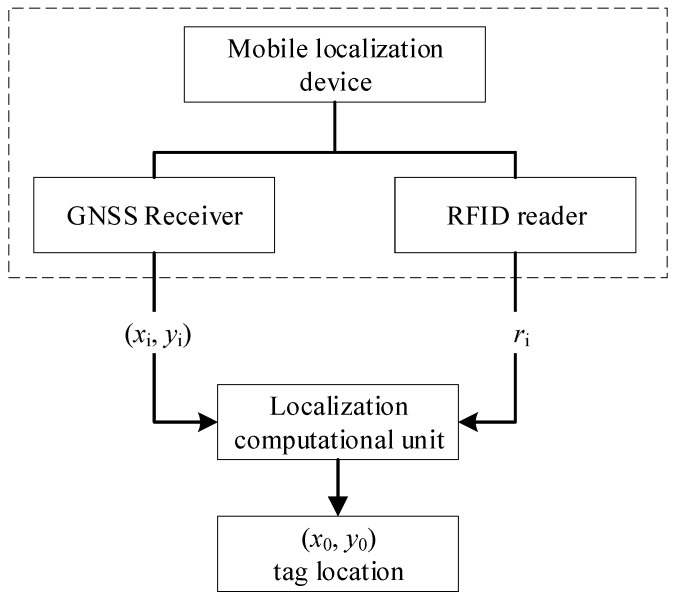
The workflow of mobile localization scheme with Global Network Satellite System (GNSS) and radio-frequency identification (RFID).

As mentioned above, mobile localization in combining with GNSS and RFID is a low-cost scheme comparing to reference tag approach and variants. In this scheme, a mobile RFID reader mounted with a GNSS receiver replaced the reference tags (for detailed information about the scheme, please refer to Reference [11]). The workflow of the localization scheme is shown in Figure 1.

In the mobile localization scheme, the GNSS receiver provides the coordination of RFID reader, which is denoted as (*x*_i_, *y*_i_). RFID reader ranges the distance *r*_i_ between a reader and target tag with unknown coordinates denoted as (*x*_0_, *y*_0_). When moving of RFID reader, many groups of value are measured. The target tag location of (*x*_0_, *y*_0_) will be computed with the trilateration algorithm.

### 3.2. Noise Influence

**Figure 2:**
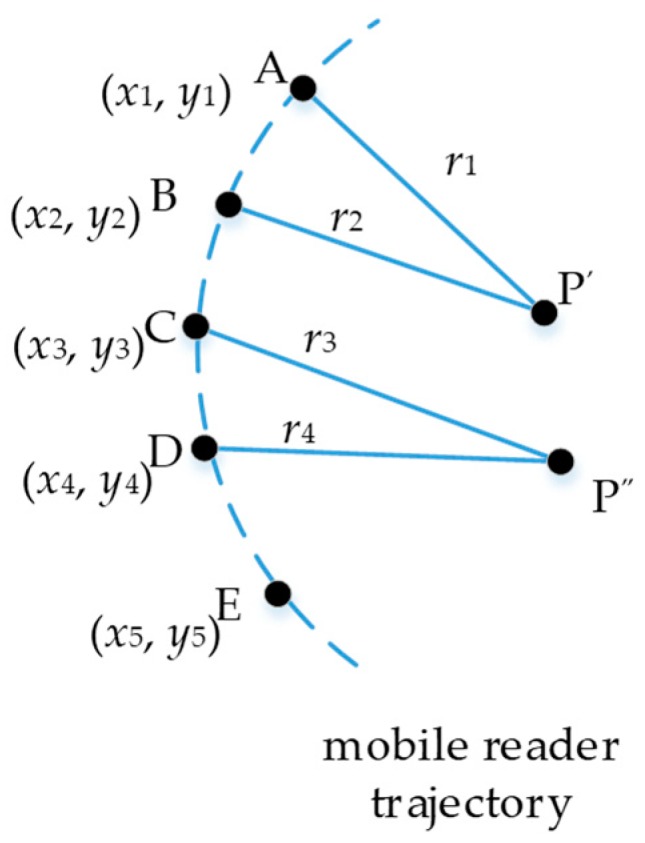
Localization noise example.

According to the mobile localization scheme, considering the five trajectory points in the mobile reader trajectory demoed in Figure 2, denoted as A, B, C, D, E, the following equations are satisfied:

(1) $${\left(x_{i}-x_{p}\right)}^{2}+{\left(y_{i}-y_{p}\right)}^{2}={r_{i}}^{2}\;i=1,2,3\ldots$$

where P is the target tag point, coordinates denoted as (*x*_p_, *y*_p_).

The intersection point can be picked up by solving the following equations at the point i and j as:

(2) $$\{\begin{array}{c} {\left(x_{i}-x_{p}\right)}^{2}+{\left(y_{i}-y_{p}\right)}^{2}={r_{i}}^{2}\;i=1,2,3\ldots \\ {\left(x_{j}-x_{p}\right)}^{2}+{\left(y_{j}-y_{p}\right)}^{2}={r_{j}}^{2}\;j=1,2,3\ldots \end{array}$$

Ideally, all results derived from all points in the trajectory are the same value, which is displayed as one overlaid point in Figure 2. However, it is always impossible owing to the existing noise data. Therefore, the different solution to the Equation (2) may be derived at different points. As the example in Figure 2, P′ is solved from points A and B, but the P″ is the result from points C and D. It is the so-called noise in mobile localization. The noise stemmed from the surrounding environment, such as the moving bus, pedestrian, the mental materials, and other signal influence materials, which are dynamic and more challenging to model [16].

The scale of the difference to the true value is changed along with different surroundings. Some of them may be mainly influenced and larger than the threshold of the true value. Therefore, we refer them as the error that can be eliminated by some mathematical model. Considering the geometry characteristic in the trilateration method, a triangular model named delta filter is engaged to model and identify the error. However, some may be small and cannot be modeled easily, which is called noise data within the threshold of the true value. Noise identification and mitigation are crucial to ensure accuracy, which has been one of the hot research topics [50]. In this paper, considering the principle of mobile localization, we proposed a RANSAC based noise mitigation method to identify and reduce these noises impact.

## 4. A RANSAC Based Noise Mitigation Method

### 4.1. Delta Filter for RFID Measurement Data

In trilateration, the situation when there is a tiny angle for mobile localization is undesirable for the mobile localization method. Unfortunately, it happens very often. A small angle means a sharp apex vertex in a triangle composed of two trajectory points and target tag point (such as ∆ABP’ in Figure 2), which reduce the accuracy of the localization method. It is similar to an ill-conditioned triangle in Geomatic survey [51], which has one of three angles bigger than 120 degrees or smaller than 30 degrees. The ill-conditioned triangle introduces unsteady accuracy. We designed a simple delta filter algorithm to identify and remove these ill-conditioned triangles.

**Figure 3:**
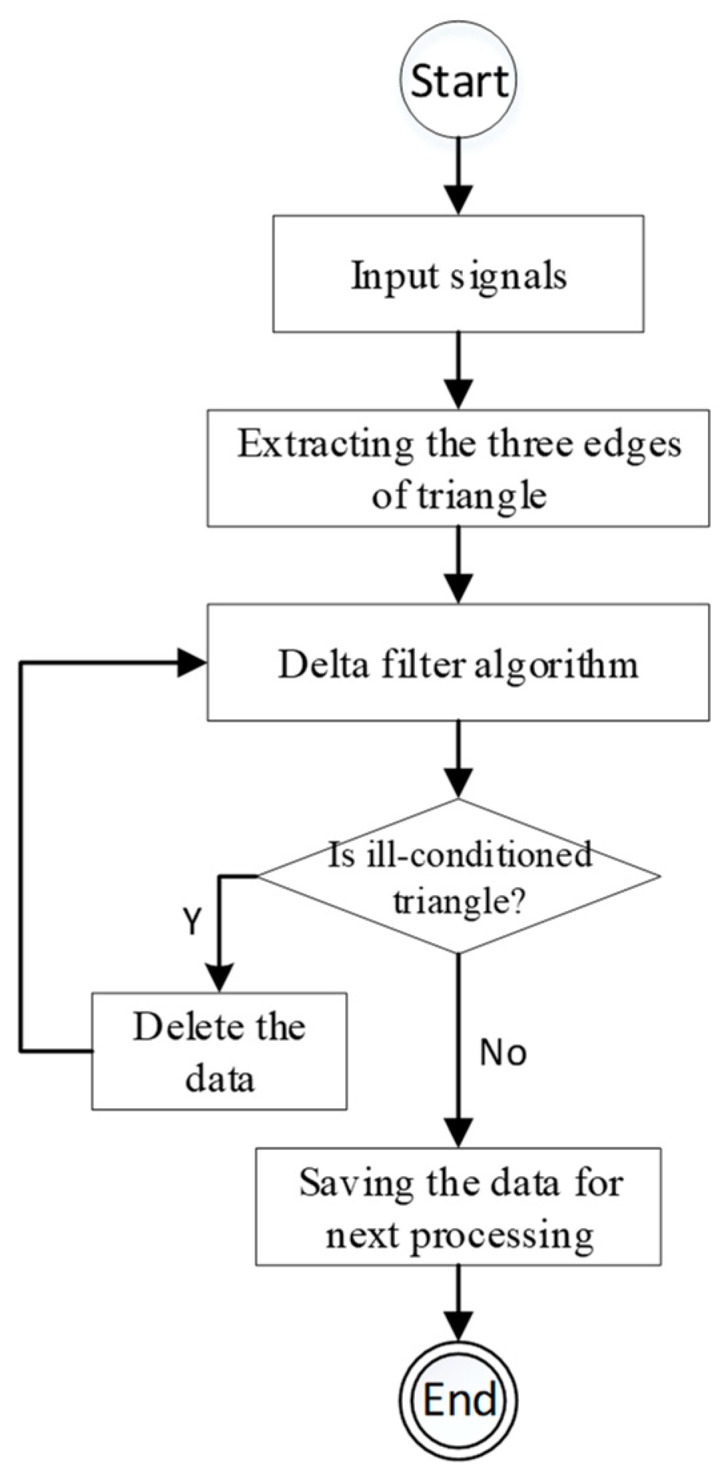
The workflow of delta filter processing.

Considering the three edges of the triangle are well-measured by a mobile device in our scheme, the angle of vertex P′ is inferred from three edges. In our delta filter algorithm, the value of the vertex angle is the essential rule for the ill-conditioned triangle, which must fall in (30, 120). The workflow of delta filter processing is shown as Figure 3. And the algorithm for delta filter is descripted as Algorithm 1. The performance of the delta filter is in the data collection phase. Cleared data with the delta filter algorithm are prepared for the RANSAC based robust noise detection.

**Algorithm 1:** Delta filter algorithm**Input:** GNSS signal data, RFID ranging data**Output:** Satisfied and well-conditioned triangle data1**set***p* = point coordinate of the new point from GNSS signal data2**set***r* = distance from RFID ranging signal data3**For each** point in saved reader location points set **do**4   **set**
*p*_i_ = point coordinate of points set5   **set**
*r*_1_ = distance between *p*_i_ and tag6   **set**
*r***_2_** = distance between *p* and tag7   **set**
*r*_3_ = distance between *p*_i_ and *p*8   **calculate** vertex angle of tag point from *r*_1_, *r*_2_, and *r*_3_9   **if** the value of the angle is within (30,120) **then**10      The *p*, *p*_i_ and tag point can build a well-conditioned triangle,11      then add the triangle data (*p*, *p*_i_, target point) to a data set for the next process12   **else**13      continuous the next loop14   **end if**15
**End for**
16Add (*p*, *r*) to reader location points set for next reader location processing

### 4.2. A RANSAC-Based Robust Noise Detection

In this section, we describe a RANSAC-based robust noise identification and mitigation method for the mobile localization scheme in smart cities, which is capable of detecting the noise data stemmed from synthetic influence including the surrounding environment and device system errors. As mentioned in the above sections, this method is more efficient than the traditional noise detection methods, such as Kalman filter, WCL, and Least Square. On the one hand, it is only used the high-reliability data with less or zero noise, which is differentiated from the concept of using the mean value of all data in other methods. Since the existing of uneven noise in the latter method, the larger noise may give more weight on tag coordinates resulting in a large error. On the other hand, the synthetic noise in mobile localization scheme is difficult to model for the traditional method. As many works of literature proposed [20,28], RANSAC-based noise robust mitigation framework for the mobile localization scheme still has two phases: Hypothesis and model verification.

#### 4.2.1. Making Hypothesis for the Mobile Localization

From the principle of RANSAC [52], the hypothesis model is the predefined fit model for original data, which inferred from the physical significance of the problem. For the mobile localization, the computational model is given in Section 3. However, RANSAC suggests a linear process [53]. Therefore, the linear of Equation (2) is the first work to define a hypothesis model.

For Equation (2) in Section 3, when the second equation subtracts the first one, it can be written as:

(3) $$({\left(x_{j}-x_{p}\right)}^{2}-{\left(x_{i}-x_{p}\right)}^{2})+\left({\left(y_{j}-y_{p}\right)}^{2}-{\left(y_{i}-y_{p}\right)}^{2}\right)={r_{j}}^{2}-{r_{i}}^{2}\;i,j=1,2,3\ldots n$$

Unfolding this equation, a linear one can generate.

(4) $$\begin{array}{c} -2*\left(x_{j}-x_{i}\right)*x_{p}-2*(y_{j}-y_{i})*y_{p}=({r_{j}}^{2}-{r_{i}}^{2})-({x_{j}}^{2}-{x_{i}}^{2})-({y_{j}}^{2}-{y_{i}}^{2})\\ i,j=1,2,3\ldots n \end{array}$$

In mobile localization, only the (*x*_p_, *y*_p_) is the unknown variable. Therefore, it is:

(5) $$m*x_{p}+n*y_{p}=1$$

where

$$m=\frac{-2*\left(x_{j}-x_{i}\right)}{\left({r_{j}}^{2}-{r_{i}}^{2})-({x_{j}}^{2}-{x_{i}}^{2})-({y_{j}}^{2}-{y_{i}}^{2}\right)}\;i,j=1,2,3\ldots$$

$$n=\frac{-2*(y_{j}-y_{i})}{\left({r_{j}}^{2}-{r_{i}}^{2})-({x_{j}}^{2}-{x_{i}}^{2})-({y_{j}}^{2}-{y_{i}}^{2}\right)}\;i,j=1,2,3\ldots$$

Transforming from Equation (5), a new equation is:

(6) $$n=\frac{-x_{p}}{y_{p}}*m+\frac{1}{y_{p}}$$

It can be written as the standard linear form.

(7) $$y=a*x+b,\;Where\;a=\frac{-x_{p}}{y_{p}},\;b=\frac{1}{y_{p}},\;y=n,\;x=m$$

Equation (7) is a linear form that is suitable for the RANSAC method. The *m* and *n* are the variables that can be calculated by the measurements. With the RANSAC method, the fitted value of *a* and *b* can be obtained. Then, the value of (*x*_p_, *y*_p_) can be achieved.

#### 4.2.2. Parameters Definition for Verification

According to the principle of RANSAC [52], the observed data should fit the hypothesis model as much as possible. The model is progressively verified by sampled data with parameters, including minimum sample points (*min_samples*), the residual threshold (*t*), and the maximum number of iterations for random sample selection (*max_trials*).

In the hypothesize-and-verify framework, repeatedly sampling subsets of the data to the hypothesis model, and then verifying whether it is uncontaminated data. Unlike all the data in the verification in the traditional regression filter method, only little data are needed for verifying the model. Therefore, the RANSAC algorithm has sufficient noise detection capability. The parameter *min_samples* means the minimum sample size of sampling subsets. Many pieces of the literature suggest that parameters should be as small as possible [20,27,28]. Therefore, for the hypothesis model in Equation (7), the value of *min_samples* is set to 2.

The residual threshold (*t*) parameter is to determine whether the observed data supports the hypothesis model. Although it is essential for model verification, unfortunately, it is difficult to define with the empirical work. Many researches proposed the determining method based on the probability distribution of observed data, such as Gaussian normal distribution [19,22] and chi-square distribution [27]. However, it is only in the high signal-to-noise ratio scenario that the RFID signal distribution is consistent with Gaussian distribution [54]. The uncertainty of noise makes it impossible with Gaussian distribution in the outdoor environment. Thus, the median absolute deviation is engaged as a machine learning software package suggested [55].

The parameter *max_trials* is one of the two stopping criteria, which means the maximum number of iterations for random sample selection. As many literatures defined [27,28,52], this parameter value can be expressed as:

(8) $$max\_trials=\frac{\log\left(1-p_{0}\right)}{\log\left(1-\varepsilon^{n}\right)}$$

where *n* is the sample size or points number in sampling. $p_{0}$ is the level of confidence that at least one of the selected minimal subsets is outlier-free. The value is often 0.95 or 0.99. In our solution, we get 0.99. The *ε* is the probability of inlier data points in the dataset, that is, the true inlier ratio. Although the fraction of inlier data ratio is unknown, we could set the value as the maximum tolerance ratio as existing literature suggested [27]. According to existing literature [20,29], RANSAC could detect more than 50% noise data dataset. Therefore, the value of *ε* is set to 0.5.

## 5. Experiment and Result

### 5.1. Experiment Setup

**Figure 4:**
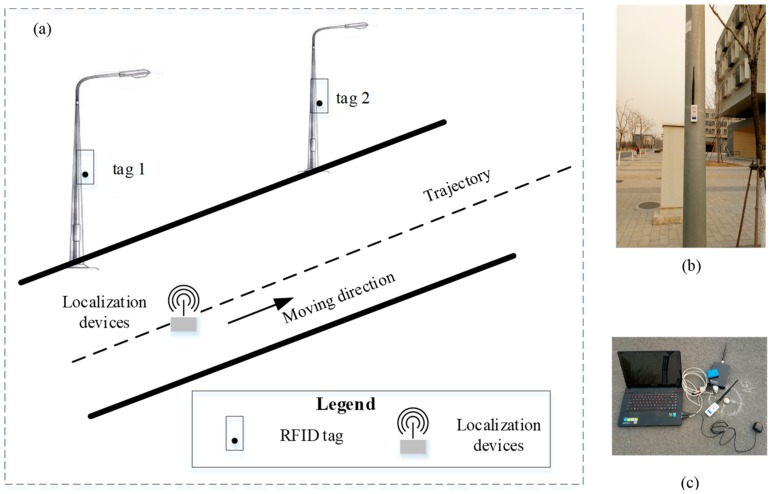
The overview of the experiment. (**a**) is the sketch of the experiment; (**b**) is the tag placement scenario; (**c**) is the localization devices.

To evaluate the performance of our approach for infrastructure assets localization in a smart city environment, we conducted experiments in a university campus. On this site, the experiments are designed as shown in Figure 4. The tags are deployed at the target, light poles. The localization devices are connected to a computer for data analysis, which is moving with a person. During the moving, the coordinates of the RFID reader and the distance between RFID reader and tags are recorded for the localization of target tags. The observed value is calculated by our proposed method mentioned in Section 4. The true value of poles was measured by the Leica TS06 Total Station. The root mean square error (RMSE) and empirical cumulative distribution functions (CDF) was engaged to measure the accuracy. To further investigate the performance of our developed approach, comparisons with existing noise mitigation methods are designed.

### 5.2. Field Test Result

**Figure 5:**
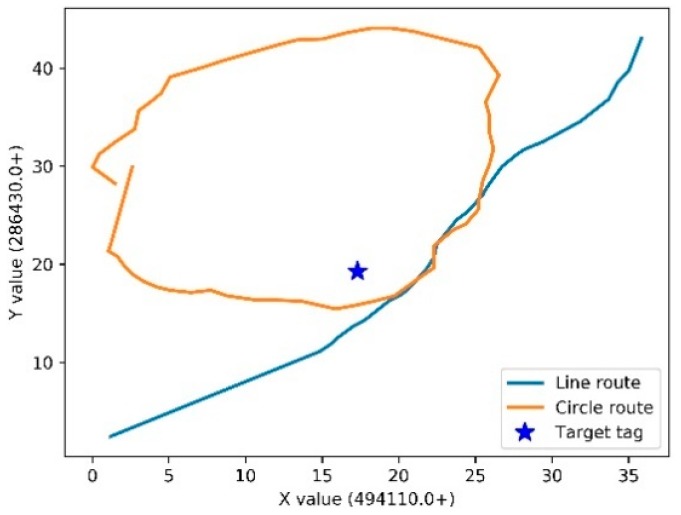
Two routes of the test.

**Figure 6:**
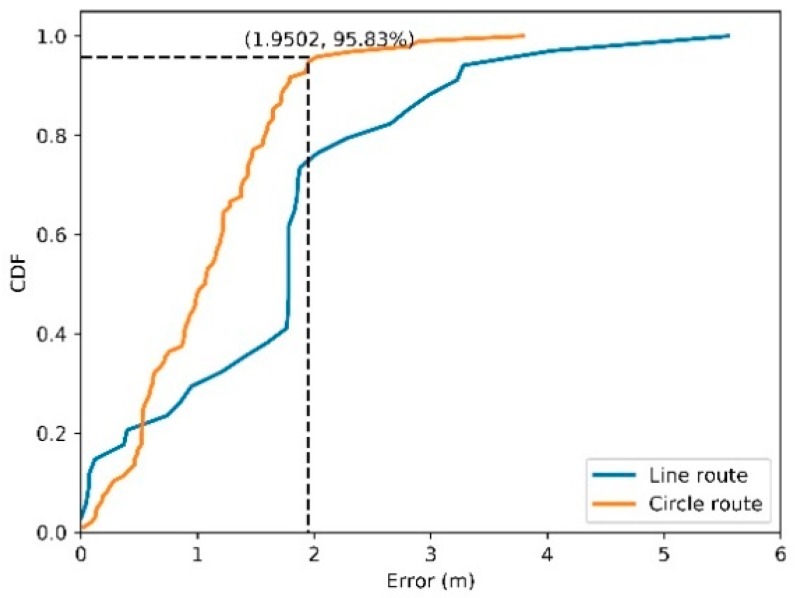
Errors of dynamic localization for two routes.

The dynamic localization experiments are conducted to verify the performance of our proposed method in a real scene. The RFID reader is held by a moving person, and the tag is deployed on target. The coordinates of the target tag were calculated along with the movement of the RFID reader. Two routes are tested as shown in Figure 5. Typically, localization error is computed based on Euclidean distance between estimated and true location. The empirical cumulative distribution functions of localization errors with the developed method for the two routes are shown in Figure 6, where x-axis represents error values and the y-axis denotes the portion of errors less or equal to corresponding values. The turning point at where the line goes flat means the stable absolute error. As shown in Figure 6, the absolute error of the circle route is 1.9502 m. The root mean square error (RMSE) indicator is engaged to demonstrate the localization accuracy for each route, which is shown in Table 1.

For the circle route, the true position of the tag (blue star) lies in the inside of the route. According to our proposed method, the delta filter was applied for the measurements.

**Figure 7:**
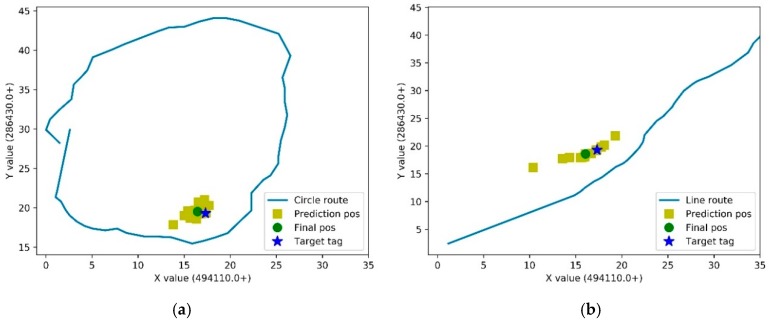
The route and tag positions. (**a**) Circle route; (**b**) Line route.

Figure 7 presents the routes and tag positions, which include prediction position (yellow square), final position (green circle), and true position of target tag (blue star). As shown in Figure 7, the true position of tag lies in the inside of the route. Meanwhile, it is on the side of the line route. For the circle route, the prediction position is evenly distributed around the target tag, which means the accuracy of every point is similar. Otherwise, it is a linear distribution of estimated positions in the line route. The possible reason may be the different noise levels along the route.

## 6. Comparison with Existing Methods

**Figure 8:**
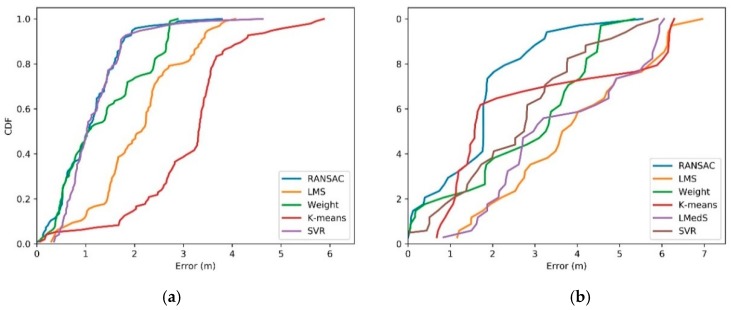
Cumulative distribution of localization error in circle route and line route. (**a**) Circle route; (**b**) Line route.

To investigate the performance of our proposed method further, we compared the results with those conducted by existing methods. These methods include Weighted Centroid Localization (WCL) [56,57], k-means [58], Least Mean of Square (LMS), Least Median of Square (LMedS) [19], and and Support Vector Regression (SVR) [16]. The RMSEs of these methods are compared in Table 1. The cumulative distribution curve of localization error was drawn as shown in Figure 8. Moreover, we put more emphasis on the effectiveness to mitigate the noise data, therefore, we ignored the improvement of RANSAC method in efficiency and accuracy comparing to other RANSAC variant algorithms.

The WCL algorithm measures the target value based on the distance between the RFID reader and the RFID tag [56]. The weights are usually proportional to the inverse of Euclidean distance, which means the weight is weak with increasing distance. Our previous research discussed this algorithm more detail for RFID localization [11]. As seen in Table 1, the WCL algorithm got a good result in our experiment for two routes. The reason is that the small distance may introduce less noise, thus a better accuracy is achieved. Otherwise, the accuracy may be lower.

The LMS is another classic estimation method. All original signals are estimated with one model, which makes it more sensitive to outliers [19]. LMS algorithm adopts the sum of different squares of all measurements. When one measurement is significantly different compared to other measurements, the sum of squares may increase sharply. The LMedS is proposed by researchers as a robust estimator, which is improved based on LMS [19]. The LMedS method uses the median of all measurement to replace the sum in LMS, which mitigates the influences by outliers. From the accuracy comparison in Table 1, a little improvement was achieved from 4.5916 m to 4.3050 m for the line route, and from 2.2975 m to 2.2624 m for the circle route.

**Figure 9:**
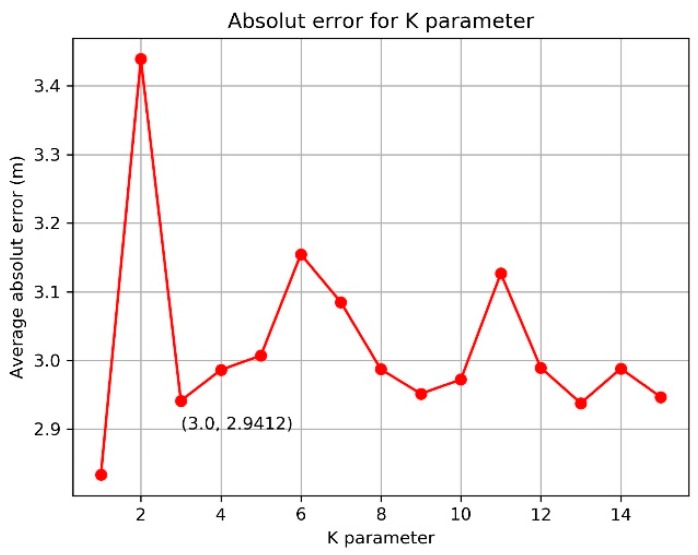
Accuracy comparison with different k parameter (circle route).

K-means is another robust estimation method, which is often used for outlier detection [58]. Its noise mitigation capability depends on parameter configuration and distance distribution. The k-means algorithm divides data as several clusters based on rulers or criteria such as distance, error distribution and position. The number of clusters is a preset parameter with experience. The different parameter results in different accuracy, as shown in Figure 9. In our experiment, k = 3 is adopted. Furthermore, the localization accuracy and noise depend on the distance trend. For the line route in our experiment, the distance between the RFID reader and tag is changing from big to small, then to big. However, in a circle route, the distance is keeping within bounds. That is, in a line route, the up and down change of distance means different accuracy of all measurements and higher possibility, including noise in measurements, owing to the relationship between accuracy and distance [19]. However, they are around the same accuracy because of a similar distance in the circle route. As we see from Table 1, K-means has a better performance in line route with a similar accuracy with WCL. Meanwhile, in a circle route, the algorithm presents weak noise mitigation with lower accuracy than other methods, owing to the even distribution of noise.

As a machine learning method, SVR method has been used in many localization approach [16,58]. The SVR method predicts the weight of each measurement to achieve good result by engaging the learning concept with training data. In our experiment, the SVR method gets the better result than other traditional method with a RMSE, 1.3573 for circle route and 3.2740 for the line route. However, they are both lower accuracy than RANSAC method. From the CDF curve in Figure 8, the SVR method has the similar accuracy with RANSAC in circle route, and lower accuracy than RANSAC in the line route.

For our method, the line and circle routes are more robust than other methods. As we can see from Table 1, the RMSE of RANSAC decreased from 4.5916 m to 2.6529 m in the line route. The percentage of errors less than 2.6 m are around 85%, as shown in CDF in Figure 8. For the circle route, the accuracy has improved from 3.2779 m to 1.2605 m. The percentage of errors less than 2.0 m is about 95%. The reasons for the better results are the filtering and RANSAC algorithm, which avoid the shortcoming in existing methods. In our method, the preprocessing of raw data by filtering algorithms, some larger measurement errors or outliers are removed. These outliers influenced the accuracy in the other methods. On the other hand, our method changes the parameters by the progressive subset sampling. That is, the regression model is adapted to the data. However, the one model is applied to all data in WCL, k-means, LMS, and LMedS, where parameters are fixed for all data. Although RANSAC is a more robust method for mobile RFID localization without any prior knowledge, it has some limitations. When the raw data has many repeated data or a very small difference between them, these noises cannot be detected. The localization accuracy may be weak. However, it can be handled easily with some preprocessing technologies. Another limitation is the noise data distribution. The more even distribution means the better result. Otherwise, the dispersed distribution of noise data increases the difficulty in detecting noise.

## 7. Conclusions

In this paper, we proposed a novel noise mitigation method integrating filtering algorithm and RANSAC, which is used to reduce noise for our previous low-cost RFID-based localization scheme. Facing the dynamic urban surroundings and large area applications, existing logic-based methods and machine learning methods have drawbacks in their dependency on extra data or devices to improve accuracy. Inspired by the higher noise detection capability of RANSAC, we expanded it with our developed low-cost localization scheme with only one mobile RFID reader, which can be used in a large area for built environments without additional hardware investment. In our proposed noise mitigation method, the delta filter is used to detect abnormal data first. To improve its robustness, it relies on the RANSAC algorithm to detect noise from data. For the usability test, two routes experiments were designed. The experimental results demonstrate its effectiveness, which has potential applications for public infrastructure assets localization in the built environment. Nevertheless, there is a limitation and room for further improvement. For example, the more repeated measurement may have a heavy influence on the regression result of RANSAC method. For the future, more filtering algorithms will be tested in preprocessing to detect anomalies and the efficiency about our approach will be horoughly verified and compared with other RANSAC variant methods. We also plan to apply our method to build environment projects and services in smart cities initiatives.

## Figures and Tables

**Table 1:** Root mean square error (RMSE) of localization methods.

	WCL	k-Means	LMS	LMedS	SVR	RANSAC
**Line Route**	3.6293	3.6957	4.5916	4.3050	3.2740	2.6529
**Circle Route**	1.6345	3.2779	2.2975	- ^1^	1.3573	1.2605

^1^ The result cannot computation owing to the computational complexity.
